# Agricultural intensification: The status in six African countries

**DOI:** 10.1016/j.foodpol.2016.09.021

**Published:** 2017-02

**Authors:** Hans P. Binswanger-Mkhize, Sara Savastano

**Affiliations:** a University of Pretoria, South Africa; b World Bank and University of Rome Tor Vergata, Department of Economics and Finance, Italy

## Abstract

Boserup and Ruthenberg (BR) provided the framework to analyze the impact of population growth and market access on the intensification of farming systems. Prior evidence in Africa is consistent with the framework. Over the past two decades, rapid population growth has put farming systems under stress, while rapid urbanization and economic growth have provided new market opportunities. New measures of agro-ecological potential and urban gravity are developed to analyze their impact on population density and market access. The descriptive and regression analyses show that the patterns of intensification across countries are only partially consistent with the BR predictions. Fallow areas have disappeared, but cropping intensities remain very low. The use of organic and chemical fertilizers is too low to maintain soil fertility. Investments in irrigation are inadequate. In light of the promising outcomes suggested by the Boserup-Ruthenberg framework, the process of intensification across these countries appears to have been weak.

## 1 Introduction

Since independence in the 1960s, Sub-Saharan African countries (SSA) have undergone exceptionally fast population growth. They also have faced rapid urbanization and some economic growth, which would have tended to increase the demand for agricultural products. In more densely populated areas, the rising population has resulted in farm sizes now close to East and Southeast Asian levels (Headey and Jayne, 2014, Otsuka and Place, 2014).^1^ This means that farmers now have to fend for their livelihood on a much reduced area, which requires rapid intensification and productivity growth. At the same time, the rising demand for agricultural commodities should be beneficial for them in terms of better market opportunities and higher prices for non-traded commodities. Both forces are leading to higher farming intensities, and possibly to higher investments and input use.

Under the theory of intensification of farming systems of Boserup, 1965, Ruthenberg, 1980a, Ruthenberg, 1980b, the BR model of intensification, both population growth and market access can lead to a virtuous cycle of intensification of agriculture: These forces lead to a reduction in fallow, higher use of organic manure and fertilizers to offset declining soil fertility, and investments in mechanization, land and irrigation. All of these have the potential to offset the negative impact of population growth on farm sizes, maintaining or increasing per capita food production, and even increase a farmer’s income, which we call the BR predictions. Population growth provides the necessity for intensification, while market access provides the opportunity.^2^ The increase in output, however, comes at the cost of an increase in labor and other inputs per hectare cultivated. The positive outcome has been realized in those tropical areas of the world where technical change has added impetus to productivity growth.

However, another outcome observed by Geertz (1963) in Java prior to the Green Revolution, was that the intensification triggered by population growth and market access was insufficient to lead to enough productivity growth to make today’s farmers better off than their parents, and that instead, they became worse off. Geertz called this process agricultural involution.^3^ Since the 1960s, biological technical change in SSA has been lagging behind the rest of the world, and so have fertilizer use, mechanization and investment in irrigation (World Bank, 2008). The question, therefore, is whether there has been agricultural involution in Africa, which was first addressed by Lele and Stone (1989), who found significant signs of involution. Have increases in farm profits per acre been sufficient to also lead to an increase in agricultural income per person, more than offsetting the decline in land per person? This is the research question that needs to be evaluated in Africa, and towards which we make a modest contribution.

The literature on agricultural intensification in Africa developed significantly in the 1980s and has resumed over the past decade. As shown in the literature review below, it generally finds that in most areas studied, intensification has progressed along the lines predicted by Boserup and Ruthenberg, and that agricultural involution is confined to a few areas. These studies typically used case studies across locations. However, Headey and Jayne (2014), using cross country data, have shown that rises in population density have been associated with reduced fallow and more intensive use of fertilizer, but not in mechanization or irrigation. That would make involution very likely, as it is hard to see how yields and farm profits per acre could increase much under these circumstances. Testing whether involution is occurring or not would require access to micro-panel data that is not yet available in Africa over a sufficiently long period.

In this paper, we instead take initial steps towards analyzing the status of intensification processes using national representative household data. They are for six African countries that have been collected under the Integrated Surveys on Agriculture (ISA) that have been imbedded in broader Living Standard Measurement Studies (World Bank, 2009) (Ethiopia, Malawi, Niger, Nigeria, Uganda and Tanzania). These national household data contain the intensification and technology variables, as well as profits and household incomes. These will generate panels of five or more years of data which will have to be analyzed in the future. In this paper, we use the cross section data from the first year of the studies. We are therefore not able to rigorously test the BR predictions. However, rigorous tests of the BR framework micro-data has to wait until panel data of sufficient length become available in order to enable an analysis of changes in farming systems that may be quite slow. Instead, we are focusing on the description of the status of agricultural intensification in the six countries, including population density, cropping intensity, fallow, irrigation and use of inputs. We then check whether there is consistency of the predictions of the BR framework with respect to these variables, and among them.

In the Boserup-Ruthenberg framework, the main drivers for agricultural intensification are population density and market access. These in turn are partly determined by the agro-ecological potential of a village, as people would have migrated more to high potential areas, such as tropical highlands, and have been able to support more children; and governments would have preferred to invest in roads and markets to take advantage of the food production potential and serve the dense population (Binswanger et al., 1993). Investments in roads and markets are likely to also depend on the strength of urban demand for food, and the distances of urban centers from the villages. In this paper, we also explore the relationship of the two drivers of intensification, population density and market access, to the agro-ecological endowment and the strength of urban demand impacting on the survey villages. In order to do so, we develop a single variable for the agro-ecological potential (AEP) of each enumeration area, and a second variable for urban gravity (UG) which reflects the economic size of the city in question and the travel time from the enumeration area to the city (see below). Clearly, these two variables are exogenous to the population density and government investments for market access, and we therefore can estimate a causal impact of these two variables on the BR drivers of intensification. The finding is that high AEP and UG have had a significant positive impact on population density of the enumeration areas and on better infrastructure and market access.

We can also estimate the total impact of AEP and UG on the various intensification variables, such as cropping intensity, fallow or the use of new seeds and fertilizers. The total impact includes the impacts via all pathways by which AEP and UG influence intensification, including via population density and market access. What we are not able to do, is to measure the components of the total impact that operates via population density and market access, and therefore the regression we present does not yet constitute a rigorous test of the BR framework.

The measure of a single agro-ecological potential (AEP) variable is based on the modeling of attainable crop yields across all agricultural areas of the globe, estimated by IAASA and FAO (Tóth et al., 2012). As a proxy for urban demand, we develop a measure of urban gravity (UG) that a particular location experiences with respect to all urban centers in the country with a current population of over 500,000 people.^4^ We use an estimate of the light emitted at night by each city that is derived from exiting light intensity measures across all pixels of the city.^5^ The light emitted by each city is assumed to be highly correlated with its overall GDP. We convert the light intensity to an urban gravity variable that is a negative exponential function of the distance of the urban area from the enumeration area (EA) in which the farmers live.

More specifically, this paper will

1. Develop internationally comparable measures of the overall agro-ecological crop potential (AEP) and of Urban Gravity (UG) in the farmers’ location.
2. Describe the degree of agricultural intensification across the countries, and across the agro-ecological zones found in these countries.
3. Estimate the causal impact of agro-ecological potential and UG on population density, infrastructure and market access, and on a range of agricultural intensification variables.

As discussed, a rigorous test of the BR framework has to await panel data analysis. Nevertheless, some of the country data allow for consistency checks to be made of the observed values with the BR predictions, and these will also be signaled.

The plan of the paper is as follows: Section 2 reviews the theory and findings about agricultural intensification. Section 3 presents the analytical framework needed to test the BR framework rigorously and to estimate the impacts of AEP and UG on population density and market access, as well as their total impact via all routes they influence. Section 4 describes how the AEP and UG variables are constructed and defines the variables for all the intensification variables used in the paper. Section 5 presents the descriptive results while section six presents the regression results. Summary and conclusions follow in Section 7.

## 2 Agricultural intensification: Theory and findings

The general model of the evolution of farming systems originates in the work of Ester Boserup, 1965, Ruthenberg, 1980a, Ruthenberg, 1980b – henceforth referred to as the BR theory or framework. In the 1980s, these ideas were summarized, partially formalized, and tested for SSA in books by Pingali et al., 1987, Binswanger and McIntire, 1987, McIntire et al., 1992. All these authors consider the evolution of farming systems, the methods of maintaining soil fertility, the technology in use and the labor input per hectare as endogenous, being influenced by the both agro-ecological and the socio-economic characteristics of the environments with which the farmers are confronted. The main driving forces of the evolution of the farming systems towards higher intensification and crop–livestock interaction are population pressures (often measured as population density) and market access, both of which define the opportunities and constraints of households in the areas.^6^ Market access consists of two factors: The external demand that emanates from the urban sector and export markets, and roads which enable farmers to reach these markets.

In low population density areas (other than the arid zone), cropping is characterized by long forest fallow systems in which the re-growth of the forest after cultivation restores soil fertility in terms of nutrients and soil structure, and suppresses weeds. Land is cleared by fire, with the ashes further increasing the nutrient content of the soil. Seeding takes place between the stumps, using a digging stick or a hand hoe. The stumps make the use of a plough impossible. Weeding is not necessary as weed seeds have decayed during the long fallow period. Farmers hold no cattle. The labor requirements for producing crops are very low. After one or several seasons of cultivation, soil nutrients and soil organic matter decline, the soil structure deteriorates, and weeds start to take over. Declining yields and rising labor requirements for weeding and land preparation lead farmers to abandon the land and open new forest areas or re-grown forests for cultivation.

If population growth reduces the availability of forests and fallow land, and if new market opportunities emerge, farmers have to intensify agricultural production. They do it in order to maintain or increase their food supplies and the income from the sale of crops. The BR effects of higher population density and improved market access in the past have led to the following impacts, which are also predictions for the future:

1. The progressive reduction in fallow length until the land is permanently cultivated, and from there onwards, to multiple cropping per year.
2. Soil fertility must be restored via the incorporation of nearby vegetation into soils, preparation of compost and/or manure, and/or artificial (inorganic) fertilizers.
3. The appearance of grassy weeds makes hand hoe cultivation much more difficult, and, as tree stumps disappear in the short fallow stage, the plough is introduced via animal draft or tractors.
4. Cultivation moves from lighter soils on mid-slopes to heavier soils in lower slopes and depressions that have higher water retention capacity, and to more fragile soils on the upper slopes.
5. Cultivation in these new areas often requires investment in land for the prevention of soil erosion, and/or drainage and irrigation.
6. Farmers and herders start to trade crop residues for cattle dung, the start of crop–livestock interaction. Eventually, farmers acquire animals and herders sometimes acquire cropland, which leads to livestock integration.
7. Labor requirements per unit of land increase for restoring of soil fertility, weeding, land preparation, for investments in land, and for the maintenance of draft animals.
8. Land rights evolve from general rights of the communities which occupy an area to cultivate in their territory to individualized property and use rights to specific plots of land. This process radiates from the homesteads to more distant areas, including land under fallows and pastures. Common property resources are progressively privatized.
9. Intensification leads to increases in yields, which is faster where new technology or irrigation is introduced, and often to the diversification from basic staples to higher value crops.
10. Value of output per acre increases, but, on account of higher input costs and/or declining farm sizes, profits per acre and agricultural incomes per households may increase or decrease.

We will analyze most of these dimensions of intensification. Because on account of population growth and/or higher input costs, profits per acre and household income may increase or decline or, as suggested by the involution hypothesis, in panel data it is possible to test for it, but not yet in this paper.

Formally, the involution hypothesis associated with population growth can be expressed as follows: Net farm income (input costs) per capita, $\frac{Y_{f}}{N}$, is by definition the product of net farm income per hectare, $\frac{Y_{f}}{L}$, and land per capita, $\frac{L}{N}$:

(1) $$\frac{Y_{f}}{N}=\frac{Y_{f}}{L}.\frac{L}{N}\text{,}$$

or in percentage change terms:

(2) $$\Delta \ln\frac{Y_{f}}{N}=\Delta \ln\frac{Y_{f}}{L}+\Delta \ln\frac{L}{N}$$

If population density is rising, then land per capita is falling, leading to a loss of income, all else being equal. Of course, the Boserup argument is that all else is not equal because households intensify production (increase output per hectare, $\frac{Y_{f}}{L}$). Thus, the extent to which net income per capita declines or rises depends on whether changes in net income per capita compensate for declines in land per capita. However, it is also necessary to account for the higher input cost, will make the income increase needed to compensate for declining farm sizes even larger. Nevertheless, a second cause of ambiguous welfare effects is that welfare is better represented by net farm income, or gross income less costs. The intensification process involves an increase in a number of costs, including labor, oxen, modern inputs and land preparation (e.g. irrigation). Even with rapid production growth, net farmer income may not rise or may actually fall.

Increases in household welfare, where they occur, are often associated with diversification of agricultural production to a broader range of high value products that are often less land intensive (e.g. fruits and vegetables) and that can be marketed through improving commercialization channels. Where rising population pressure and market access lead to increased specialization, and where agricultural technology adoption and input use increase, there may be a beneficial diversification into rural nonfarm activities.

In contrast to positive intensification processes, under very high and rising population density and poor policy, institutional or agro-ecological environments, intensification could lead to involution and the diminution of economic and social well-being, and environmental degradation. Geertz (1963) characterized involution as a situation in which increasing demand for food is met by highly labor-intensive intensification, but at the cost of very small and decreasing marginal and average returns to inputs. Because there still is relatively little landless labor in SSA, the extra work would often fall on family workers, rather than being supplied by landless workers, as in Asia. Of the 10 cases of very high population density in SSA studied by Turner et al. (1993), there are signs of involution in the humid tropical areas of Imo State in Nigeria, and in the Usambara mountains of Tanzania, where special rules inhibit erosion control because it can jeopardize access to land for women.^7^ Based on macro- rather than micro-data, Lele and Stone (1989) also suggest that a significant share of the intensification observed in SSA was already showing signs of involution by the mid-1980s. This means that the conclusions from aggregate data are more pessimistic than from case studies.

Headey and Jayne (2014) find that agricultural intensities in much of African agriculture have reached the stage of permanent cropping. Most of the literature is consistent with the theory of intensification, in Africa, as well as elsewhere. Baltenwick et al. (2003), in an analysis of 48 sites in 15 countries of Africa, Latin America, and Asia, find that the forces of population density and market access transcend national and continental specificities and applied well across the study sites in all three continents. Their reviews, following McIntire et al. (1992), focus especially on crop–livestock integration and confirm the general trends and more detailed findings of these authors.

The papers in Pender et al. (2006) report studies of strategies for sustainable farming systems in the East African Highlands, focused primarily on low to medium potential areas. The selection of areas of lower agro-ecological potential also implies a bias in the results, this time against the BR effects, as in lower potential areas the work and investment incentives are likely to be lower than in higher potential areas. They find similar corroborating evidence for the general impacts, again with variations which will be discussed in subsequent sections of this paper. They emphasize that intensification is progressing especially well where vibrant markets are nearby. Much earlier, this had been found to be true in a case study of the agricultural history of Machakos district in Kenya, where the output demand from Nairobi played an important role (Tiffen and Mortimore, 1992). Moreover, the opportunities of earning income in Nairobi provided resources for investment in Machakos district. Clearly, urban centers present both market and trade opportunities, which point is important in interpreting the results in this paper. Finally, Turner and Fischer-Kowalskic (2010), in a tribute to Boserup’s 100th birthday, find that the Boserup framework has held up well.

Headey and Jayne (2014) used FAO data covering recent decades (1977–2007) from FAOs regular reporting and from their periodic agricultural censuses to study the process of agricultural intensification in countries from Asia and from Africa.^8^ As discussed in the modeling section, their panel data of countries allowed them to overcome the endogeneity issues associated with the response of population density to agro-ecological potential and urban gravity by using the fixed effects model.

They find that, in line with the BR model, agricultural intensification is an important mechanism to offset declining farm sizes in both Asia and Africa. In response to declining farm sizes, in Asia yields grew rapidly, while this response is absent in Africa. “In Africa, we observe no response of yields to land constraints over the short run, nor any growth of modern inputs such as fertilizers or irrigation. Instead, we observe increased cropping intensity driving around half of the growth in total crop output per hectare. This would appear to be an unsustainable intensification path given the implied mining of nutrients, and the more limited prospects for low cost irrigation investments, at least in many high density African countries.” (Headey and Jayne, 2014, p. 31). These results suggest that the full set of intensification processes discussed in this section have hardly occurred in Africa, which means that the BR model is only partially supported, a conclusion that is also reached in this paper.

## 3 Analytical framework

The analytical framework has to be able to measure the causal impact of population density, infrastructure and external demand (urban or export demand) on the various intensification variables. $H_{j}$ stand for the vector of intensification variables of an enumeration area j (EA, usually a village); let the $X_{\mathrm{ij}}$ variables stand for the drivers of intensification in ${\mathrm{EA}}_{j}$ i.e. population density, an indicator of access to infrastructure such as roads, and let $Z_{j}$ be a vector of other conditioning variables for ${\mathrm{EA}}_{j}$.

Then the correct equation for testing the BR hypothesis is

(3) $$H_{j}=\alpha +\beta_{1}X_{1j}+\beta_{2}X_{2j}+\gamma Z_{j}+\varepsilon_{j}$$

Eq. (3) relates the intensification variables to the drivers of intensification as identified by BR.

The critical coefficients for testing the BR framework are the β coefficients, which should be greater than zero. However, because the unobservable error term $\varepsilon_{j}$ influences both the X variables and the intensification variables H, the β coefficients would be estimated with unobservable variable bias. Examples are specific potentials to grow high quality coffee or fruit, special locational advantages such as proximity to water sources that could be used for irrigation or proximity to ports, or even cultural factors. Many of these factors are unobserved or unobservable and cannot therefore be captured as Z variables. Panel data are therefore required to rigorously test the BR framework.

However, we only have cross section data for each of the countries. These descriptive data can be used to check whether the levels of the various intensification variables are consistent with each other. For example, if cropping intensity has already reached 100% and there are no longer any fallow periods, soil fertility must be restored via the application of organic manure and/or chemical fertilizers. If the proportion of farmers using these techniques is low, then these variables have not responded as expected under the BR framework. Alternatively, if population density and cropping intensity are high, substantial irrigation investments should have occurred, but if they are very low, one of the BR predictions is not satisfied. The descriptive section below performs this analysis.

Over their history, areas of high AEP have attracted more migration than those with low AEP, they have been able to sustain higher population growth and therefore they are likely to have higher population densities. Recognizing the agro-ecological potential of an area, governments and communities would have been more likely to invest in infrastructure that provides access to markets. Similarly governments of urban centers with significant agricultural demand would also have been induced to invest in infrastructure. To test whether these dynamics have been in place, later in the paper we develop measures of agro-ecological potential (AEP) and urban gravity (UG) which can be a proxy for the demand pull of cities, and other influences on rural areas. Our AEP and UG are exogenous to the intensification variables and their impact can therefore be estimated via Eq. (4) without giving rise to unobserved variable biases.

(4) $$H_{j}=\alpha +\delta_{1}{\mathrm{AEP}}_{j}+\delta_{2}{\mathrm{UG}}_{j}+\gamma Z_{j}+\varepsilon_{j}$$

The δ coefficients will then estimate the sum of the direct impacts of AEP and UG on the intensification variables, as well as the indirect effect via their impact on population density and market access. If these are positive, then either the direct or indirect effects, or both, have been at work, and the regressions therefore do not reject the BR predictions. If on the other hand they are negative, it is likely that the BR predictions for the respective variable cannot have been realized. That means that zero or negative coefficients of AEP and UG can be interpreted as an absence of the respective BR effect. On the other hand, a positive coefficient could have been either a direct effect of AEP or UG, or an indirect one via their impact on population density or infrastructure.

The dependent variables are therefore as follows: Population density; distance to the nearest road and the nearest markets; cropping intensity, defined as gross cropped area per net cropped area; the proportion of area currently fallowed and fallowed in the past; the proportion of net crop area irrigated; and the proportion of households using different technologies that enhance yields – high yielding varieties, organic manure, fertilizer, or pesticides. Equations are estimated for each of the dependent variables, and in double logarithmic form. Because we want to analyze intensification in SSA, the country data are pooled and a country dummy is included to account for country-specific fixed effects.

## 4 Definition of the variables used and descriptive statistics

### 4.1 Agro-ecological potential per hectare

We calculate the agro-ecological potential from the currently available Global Agro-Ecological Zones (GAEZ) data portal^9^ of the International Institute for Systems Analysis and the Food and Agriculture Organization (Tóth et al., 2012).

For each 5 arc-minute grid cell of agricultural land of the World, the data set uses crop models to calculate *agro-climatic yields*, for 280 crops and land-use types.^10^ These are progressively aggregated to 49 crops. Agro-climatic yield takes into account climate-related constraints and uses and optimum crop calendar. GAEZ then calculates *Agro-ecological suitability and productivity* that takes into account the grid-specific soil and terrain conditions and fallow requirements.^11^ Because the crop yield estimates that have been used in computing AEP include the known impacts of soil degradation, today’s estimates are possibly a slight underestimation of past AEPs. However, much of the AEP is explained by innate characteristics of the soils that have not changed and a relatively stable climate over the past. Therefore, the current and past agro-ecological suitability and productivity are likely to be highly correlated.

A limitation of the proposed AEP measure has to be signaled: Population density, market access and intensification variables that are observed today reflect not just the potential today, but past potentials at the time that public investment and migration decisions were made. But the AEP measure reflects international prices for three very recent years, and the present cropping pattern, and therefore are AEPs for the current period. When analyzing the influence of the AEP on current farming systems variables, such as cropping intensity, value of production or input use, the current AEPs are the right variables to use. However, when we analyze the impact of AEP on population density and road investments, the AEP for a prolonged past period should be used, for which we do not have cropping pattern information. The current AEP is likely to be highly correlated with past AEPs, so we also use the current AEP instead.

We use the data on “*Potential yield*” that does adjust for fallow requirements. GAEZ contains potential yields for 28 crops, however we use those 15 for which international prices are available. These are wheat, rice, maize, barley, millet, sorghum, white potatoes, cassava, soybean, coffee, cotton, groundnut, banana, sweet potatoes, and beans.^12^

GAEZ presents potentials yields for low, medium and high input levels, of which the current values at intermediate level^13^ are the most appropriate for the proposed analysis: “In the case of intermediate input/improved management assumption, the farming system is partly market oriented. Production for subsistence plus commercial sale is a management objective. Production is based on improved varieties, on manual labor with hand tools and/or animal traction and some mechanization. It is medium labor intensive, uses some fertilizer application and chemical pest, disease and weed control, adequate fallows and some conservation measures.” (Tóth et al., 2012, p 18). In light of the limited irrigation in Africa, we are using the data for the rain-fed category. To summarize, we will use the *agro-ecological level for the current climate conditions at intermediate levels of input use under rain-fed conditions.*

The data in the GAEZ system is for the potential yield of individual crops. However, we want to characterize the aggregate agro-climatic potential in the communities being analyzed. Therefore, we need to assign a value to each of the potential crop yields. In order for the calculations to be comparable across countries, we first converted the yields into dollar values using average world market prices for the past three years during which the first rounds of the LSMS-ISA studies were carried out. The commodities include the 15 crops mentioned above, for which we have found international price data.^14^

To get a unique value for the AEP of a location, we aggregate the individual potential crop values into an aggregate potential crop value. This is best done by using as weights the proportion of each crop in the crop mix being produced in the enumeration areas or close to them. We use the average cropping pattern across all households in the EAs as weights to aggregate the potential crop values into the overall agro-ecological potential of the EA. For the aggregation of the potential crop values to AEP, we only take into account the value of the main product, and not any by-products.

Let $S_{\mathrm{iz}}$ denote the average share (across farmers j) of crop i in the ${\mathrm{EA}}_{z}$ and let $A_{\mathrm{ijz}}$ be the area under crop i of famer j (i = 1….M, j=1…N). The denominator in Eq. (5) is the total area under crop i in ${\mathrm{EA}}_{z}$ divided by the total cropped area in ${\mathrm{EA}}_{z}$.

(5) $$S_{\mathrm{iz}}=\frac{\sum_{j=1}^{N}A_{\mathrm{ijz}}}{\sum_{i=1}^{M}\sum_{j=1}^{N}A_{\mathrm{ijz}}}$$

Let $P_{i}$ be the international price of crop i. And Let $X_{\mathrm{iz}}$ be the agroecological potential of crop i in the EA j. Then, the agro-ecological potential in the EA z is

(6) $${\text{AEP}}_{z}=\underset{i}{\sum }S_{\mathrm{iz}}P_{i}X_{\mathrm{iz}}$$

We want to stress here that our estimate of the AEP may not adequately capture the “true” underlying AEP, and that the latter is therefore estimated with error.

### 4.2 Agro-ecological potential per person

In this study we use two measures of population pressure: the traditional population density (persons/ sq. km), and what we define the agro-ecological population pressure or the agro-ecological potential per person computed via Eq. (7)

(7) AEPP=AEP×100/PD

We have developed this new measure to take into account the vast differences in agro-ecological potential across EAs, regions and countries that are not captured by the traditional measure of population density.

As the population for each EA has not been collected in the LSMS-ISA surveys, we use the data for rural population density collected by the Harvest Choice project^15^ which are disaggregated to the level of communities contained in the EAs of the LSMS-ISA. This external variable includes farmers and people who are not engaged in agriculture, and since peri urban EAs are likely to have a higher non-farm population, the overall population pressure computed according to Eq. (7) for peri-urban areas will most likely go down.

### 4.3 Urban gravity

We follow Henderson et al., 2009, Gallup et al., 1999, Kiszewski et al., 2004 in using the measures of intensity of light emitted which is available for each pixel on earth. While light intensity is not a direct measure of economic activity, it is highly correlated with it.^16^ A great advantage of light intensity data is that they can be used for cities for which GDP data are unavailable, as for most cities in Africa. The data for light intensity come from the Defense Meteorological Satellite Program (DMSP) of the National Geophysical Data Center.^17^

To measure the aggregate emission of light at night from a city, the light intensities of each urban pixel are aggregated over all pixels of the city. The light intensities of the cities are converted to urban gravities (UG) by weighting them by travel time in hours to the EAs, using a negative exponential function (Deichmann, 1997). We then aggregate the resulting UGs to a national UG, separate for each enumeration area, by summing it over all cities in the country or across the border of neighboring countries with population above 500,000.^18^ We adjust the light intensity of cross-border cities by the composite index of the difficulty of movement of people, goods and information across the respective borders, using the higher difficulty of cross-border movement of the two respective countries. The result is the aggregate UG to which each EA is exposed.

As in the case of AEP, we assume that today’s urban gravity is correlated with UG over the past, during which migration, fertility, infrastructure investment decisions were made, and therefore the coefficients of today’s urban gravity capture both current and past impacts of UG. Since urban populations and incomes have changed very rapidly over the past decades, the errors in variable problem associated with past UG being imperfectly correlated with current UG is more severe than in the case of AEP. Again, for the intensification variables that changes more quickly over time, the problem will be less.

### 4.4 Public infrastructure

We used distance to the main road as a proxy of public infrastructure, and also included distance to nearest major market (which is an additional measure of market access embedded in the concept of UG). Both variables are included in the set of GEO variables collected under the LSMS-ISA project by means of households’ GEO coordinates. The former is the distance in kilometers to the nearest trunk road, while the latter is the household’s distance to the nearest major market.

### 4.5 Owned and operated land

There are two measures of plot sizes in the data, the area reported by the farmers, (the self-reported area), and the area measured by the enumerators using GPS. The measured areas are available for a large share of plots, but not for all of them. For the missing areas that would correspond to an estimate via GPS, regression analysis was used to relate self-reported area to area measured by GPS for the households that had both measures. Following Kilic et al. (2013), the estimated regression coefficients were then used to impute a predicted GPS area for plots with only self-reported areas.

Operated area is defined as owned area, plus rented in area, minus rented-out area.

### 4.6 Land use intensity

The cropping intensity (CI) of cropped land is used, rather than Boserup’s and Ruthenberg’s R-value. This is because in most countries fallow rates are now very low, and they are no longer in the transition from long or short fallow systems to permanent agriculture. The R-value is best suited for these earlier stages.^19^ Cropping intensity takes account of multiple cropping, which is the use of the land for more than one crop a year. Cropping intensity CI is defined as

(8) $$\mathrm{CI}=\frac{G}{N}$$

where G is gross cropped, the sum of the areas cropped in the main season plus the areas cropped in the second season, and N is net cropped area, the area cropped in the main cropping season. If there is only single cropping, CI is 1. It rises to 2 when all cropland is used in both seasons, and can go higher when some land is used more than 2 times in a year. The cropping intensity is calculated as the mean over households in an EA, while the population density is the mean over communities, as defined in the Harvest Choice data sets.

### 4.7 Irrigation and technology variables

For irrigation, we use the share of cropped land that is irrigated. Data on inputs and outputs are collected at the plot level, which is a subdivision of the parcel. The data do not contain the area of each plot. Because different plots may use different inputs and techniques, this means that we cannot estimate area under a particular technique in this data set. Instead, we have to focus on whether a farmer does use, or does not use, a particular technique. We estimate the proportion of households in each EA that are using improved seeds, chemical fertilizers, organic manure and pesticides.

## 5 Descriptive results

### 5.1 Agro-ecological potential, AEP per person, and urban gravity

**Fig. 1:**
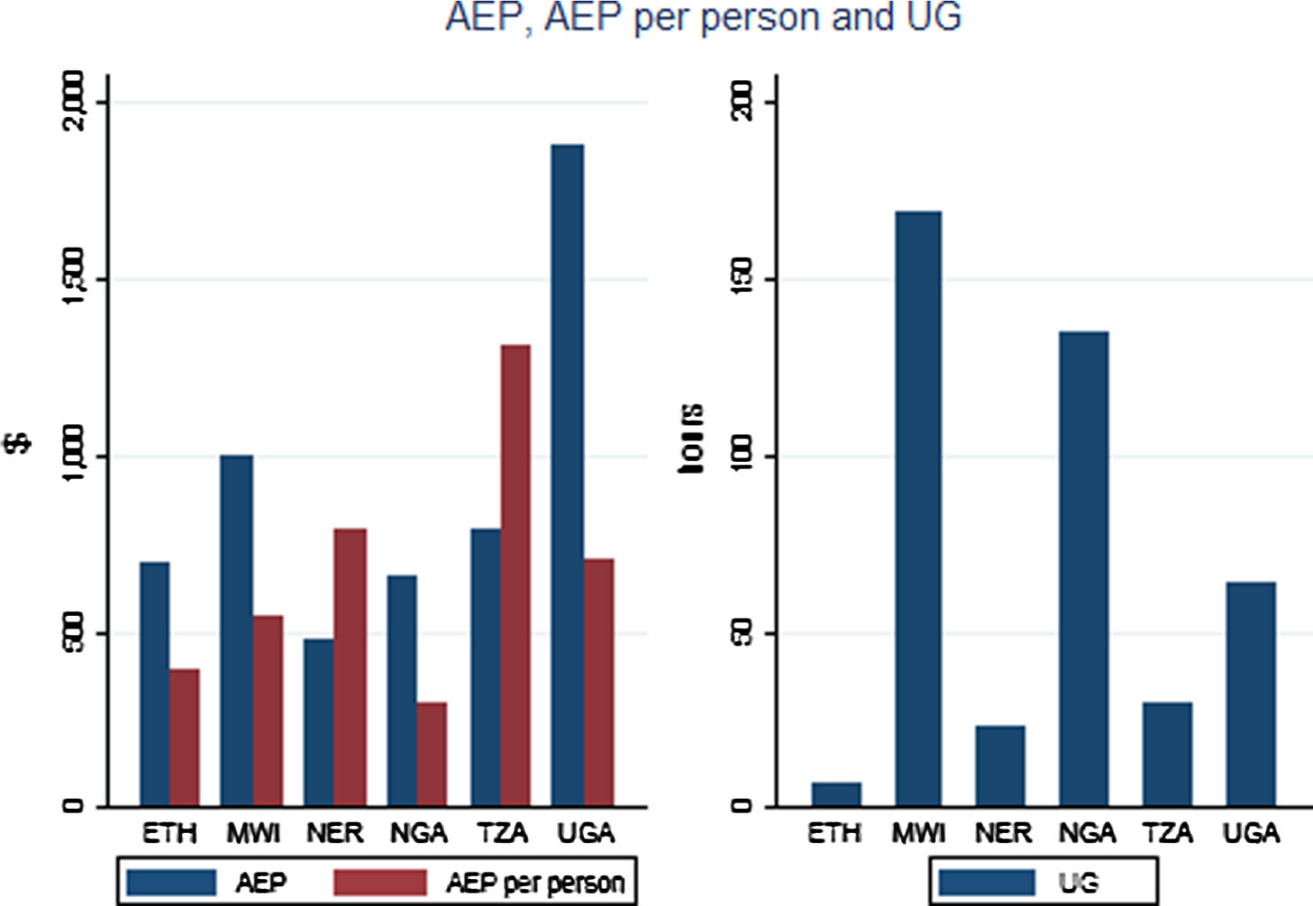
Agro-ecological potential, agro-ecological population pressure and urban gravity.

**Map 1:**
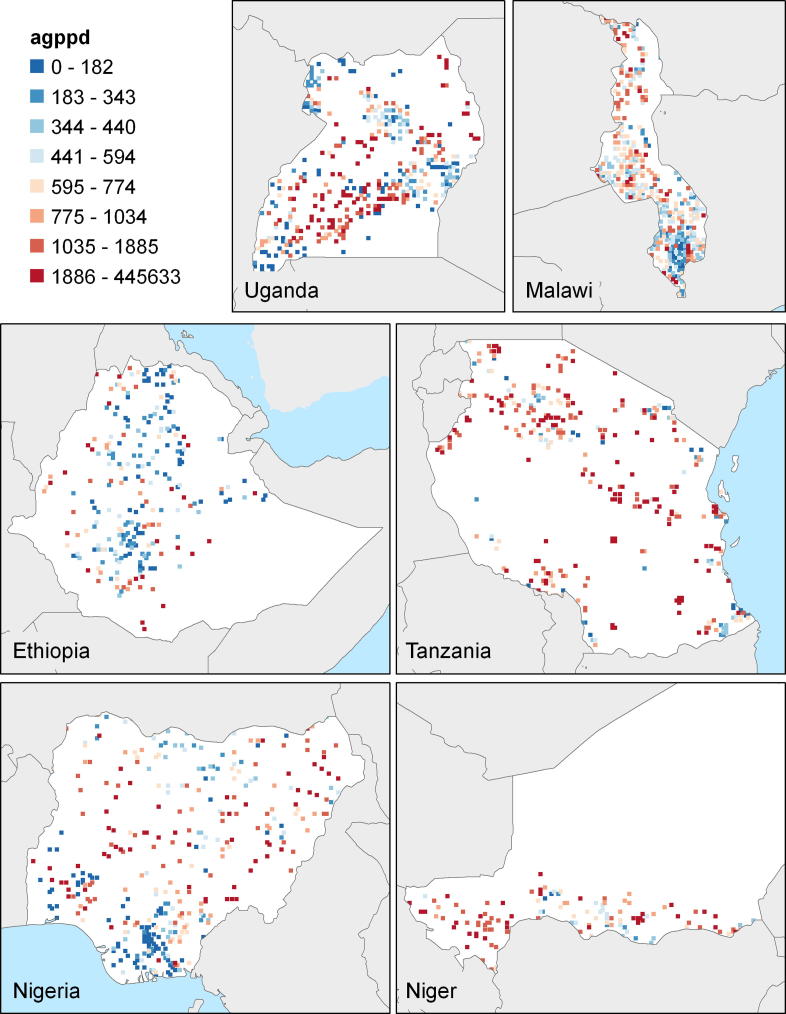
AEP/ha for the enumeration areas in of each of the six study countries.

In Table 1, Row 1, we see that the average AEP per ha across all the countries is 740 dollars per ha, evaluated at international commodity prices prevailing between 2005 and 2008.^20^ The totals across countries are population weighted. From Fig. 1, it is clear that it is the highest in Uganda, because of its good climate conditions,^21^ and the lowest is in Niger, in the very dry Sahelian zone. Map 1 also illustrates that high potential areas are most prevalent in Uganda and Central and Southern Malawi. In other countries, it is mostly light green^22^ areas, with potentials between 478 and 786 dollars per ha, rather than the darker green areas with higher potential. In Ethiopia and Nigeria, there are also many brown areas that have low potential, mainly in the dry northern parts of each of these countries. In Niger, low potential areas dominate in the entire country.

In the second row, the AEP/km^2^ has been divided by the rural population density to arrive at the AEP per person. Across all the countries, it is only $394.^23^ Fig. 1 shows that there are many reversals between AEP per ha and per person: Tanzania has the highest AEP/person at 1314$, almost twice as high as that of Uganda, a reversal with respect to AEP per ha which is on account of them having the highest and the lowest rural population densities among the countries considered. Given its dry climate, it is surprising that Niger has the third-highest AEP/person. This is on account of its high operated area per farm (Table 3) and its low population density.

Average distance of households to the nearest tarred road across all countries is 15 km, while to the nearest market it is much higher, at 66 km. Distances to roads are the lowest in Uganda, at 8 km, followed by Malawi, which also has the lowest distance to markets. The farthest distances to markets occur in Nigeria and Tanzania, at 70 km. That Nigeria, among the highest per capita income countries, should do so poorly in market access, suggests that they may have used larger markets as a reference, while Malawi may have chosen very small markets. In the regression analysis, we use the log of the variables and also include a country dummy, so that only the within country variation is used to estimate the relationships to the dependent variables, and the differences in definitions therefore are of little relevance. Among the EAs, the distances to roads and markets vary little, suggesting that most of the variation is associated with the countries, rather than the agro-ecological potential.

**Map 2:**
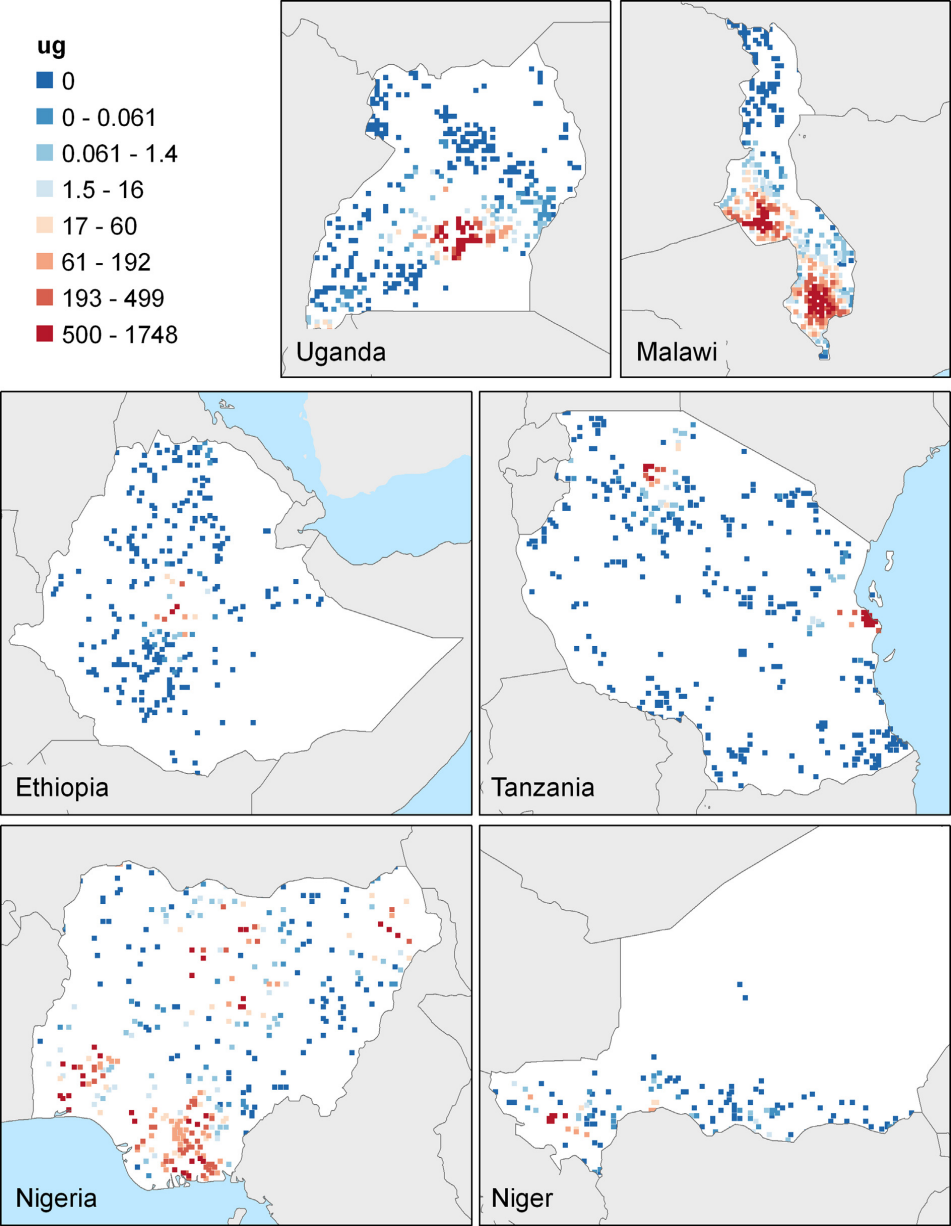
Urban gravities for the six countries.

Fig. 1 also shows the Urban Gravities for the six countries that not only reflect light intensities of cities, but also travel time. The distribution of UGs across the countries are shown in Map 2. Urban gravity is the highest in Malawi, at the value of 169, and the concentration of red dots suggests that UG is high near the urban centers of Blantyre and Lilongwe, but then tapers off quickly in the north. Then comes Nigeria, where the highest UGs are in the south, and much lower ones in the north. UG is the lowest in Ethiopia at only 7.

Table 2 shows that the rural poverty rate is the highest in Tanzania (92%) and the lowest in Niger (41%). Tanzania has not been able to take advantage of its high AEP per person to foster sufficient agricultural growth to reduce poverty. Nor has the low AEP per ha resulted in high poverty in Niger. In the other countries, the poverty rates vary between 52 and 75%.

### 5.2 Land and land use intensity

**Fig. 2:**
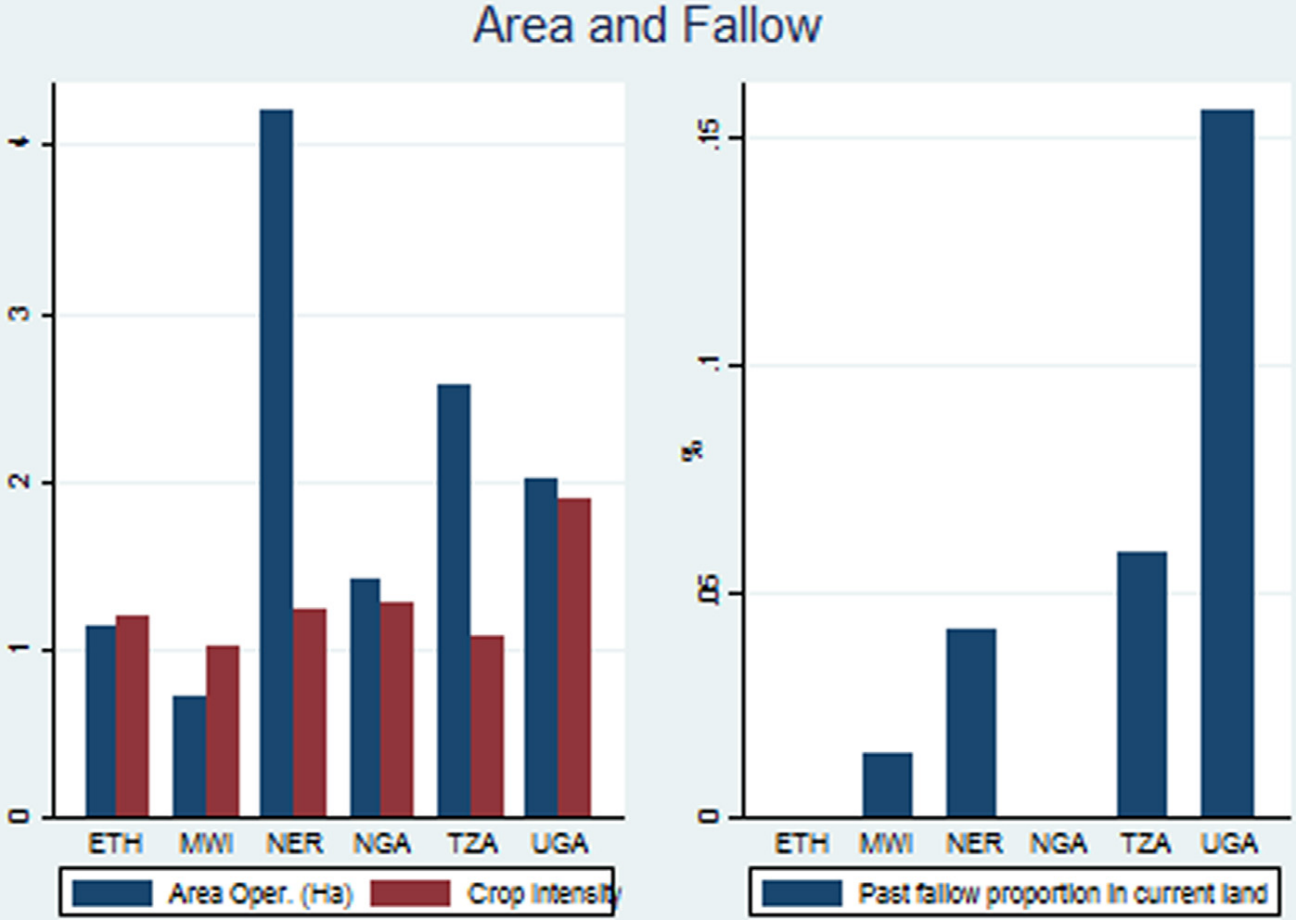
Area operated, crop intensity and fallow.

Area operated per household is owned area plus rented in area, less rented-out area. Across countries, it is on average 1.57 ha per farm (Table 3). It varies from the lowest in Malawi, at 0.74 ha, to the highest in Niger, at 5.1 ha (Fig. 2). Malawi’s AEP per ha is twice the one in Niger, which partly compensates for its low operated area. What is surprising is that Uganda, one of the high population density countries, has an operated area quite close to Tanzania’s 2.4 ha. Since Tanzania has a much lower population pressure, we would expect farm sizes there to be significantly larger. It appears that Tanzanian farmers are unable to make use of the larger land endowment per person, perhaps because they are labor constrained and unable, or unwilling, to make the investments required for animal draft or tractor plowing that would allow them to operate larger areas.

Cropping intensity is gross cropped area divided by net cropped area. It is greater than one in all countries, therefore the stage of permanent cropping has been reached everywhere. Cropping intensity is especially low in Malawi (1.01) and Tanzania (1.07): For Tanzania this is not a surprise, as is AEP per person is by far the highest in the sample of countries, indicating a low population pressure on the agro-ecological resources. However, in Malawi it the AEP per person is less than half that of Tanzania, yet its cropping intensity is the lowest among the six countries. The BR model suggests that Malawi’s high population pressure would have led to high land and irrigation investment, allowing for high cropping intensities. We therefore find another inconsistency with the predictions of the BR framework. Crop intensity is by far the highest in Uganda, at 1.89, which is on account of the bimodal rainy season. The other countries have cropping intensities between 1.19 and 1.23.

In light of permanent cropping, on average the rate of fallow in the six countries is only 1.2%, and therefore fallow can no longer contribute to soil fertility maintenance and restoration. It is clear that the high population growth rates and growth in urban demand have virtually eliminated fallows in the countries. The highest proportion of land under current fallow is found in Tanzania, at 7.5%. While that is consistent with Tanzania’s low population density and AEP per person, one would have thought that Tanzanian farmers could make more use of fallow to restore soil fertility. The lowest rate of fallow is in Nigeria, at only 0.1%. Past fallow rates are derived from the data on whether a plot had been fallowed in the year before the current year. For the four countries where we have the data, current and past fallow rates are similar.

### 5.3 Irrigation and technology

**Fig. 3:**
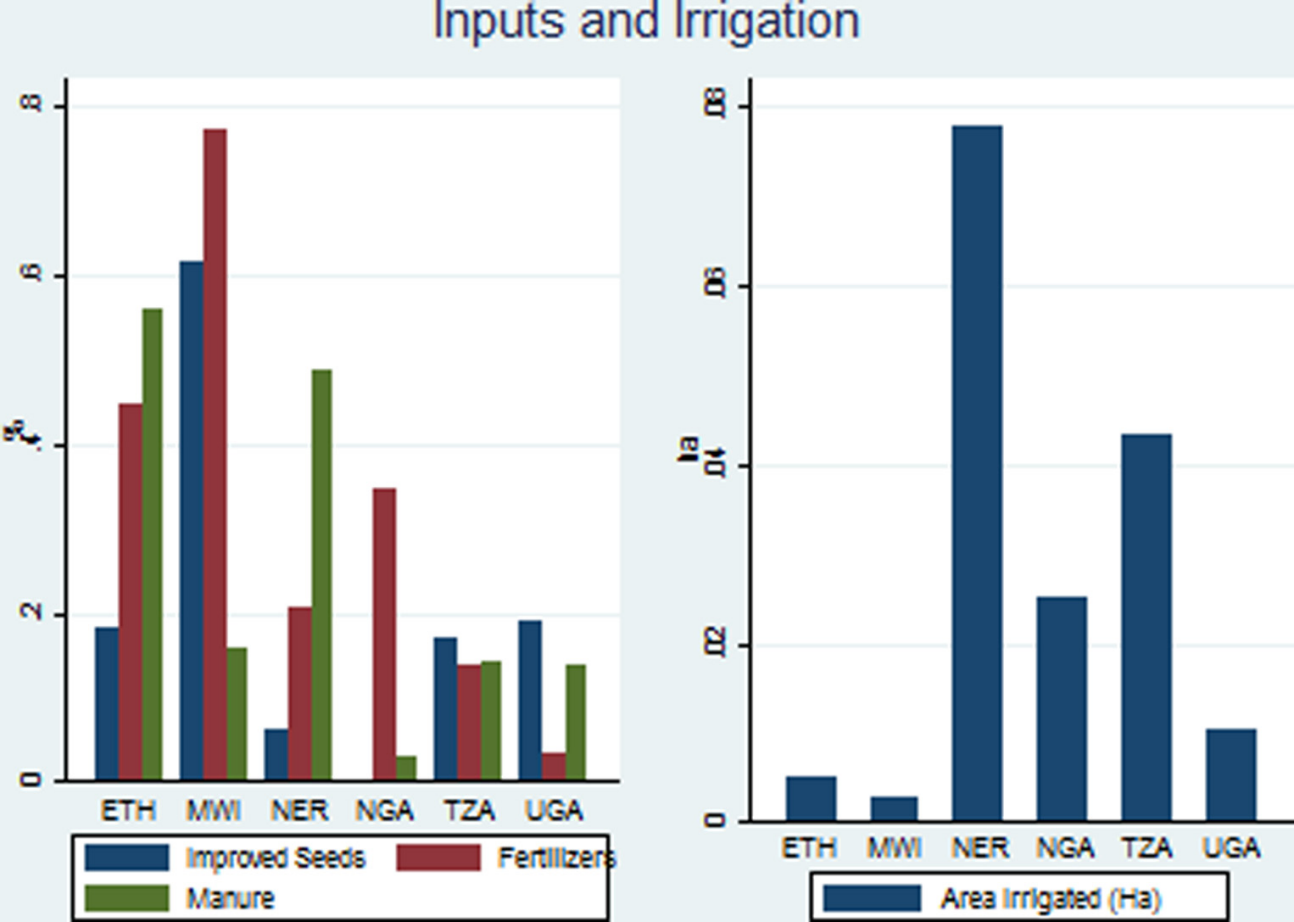
Input use and irrigation.

Across the six countries, the average area irrigated per farm is only 0.03 ha, and the share of irrigated area in total area is only 4.4% (Table 4), which in Ethiopia, Malawi and Nigeria appears to be inconsistent with the low AEPs per person observed. Surprisingly, the area under irrigation is higher in Tanzania, at 0.045 ha compared to Malawi, at 0.030 ha (Fig. 3). Given the previous discussions, this is particularly inconsistent with the BR hypotheses.

On irrigation, we also have the data by agro-ecological zones across the countries. (Online Annex). The area of land irrigated is by far the highest in the warm arid areas (0.11 ha). This is not surprising because the payoff to irrigation is higher, the dryer the climate. In all other climate zones, it is around 0.01–0.05 ha. This is also not surprising in the cool or warm humid and sub-humid areas, because the payoff to irrigation is lower in such areas than in more arid zones. What is surprising is that the cool and the warm semi-arid tropics have such low irrigation levels, as here the payoffs to irrigation are higher than in more humid areas. Irrigation, with the promise of a secure crop in the first season and a crop in the second season, should long have been a favored investment for farmers in these zones. Even if groundwater resources in Africa are less than in South and East Asia, for some farmers, they are still available. Many of these could probably have used bore-wells to install irrigation.

That irrigation, even in the semi-arid and arid zones where payoffs to irrigation are very high, is so low despite growth in population and urban demand, suggests that farmers have not responded to these trends by increasing irrigation, as the BR framework would predict. Is it possible that this lack of response is caused by exceptionally poor availability of groundwater, which farmers might have tapped via bore-wells?

Except for Malawi, the proportion of households using improved seeds is less than 18% of the households. Malawi is doing by far the best, at 61% of households. It also has the highest proportion of households using inorganic fertilizers, at 76%. Given its high population pressure, this is consistent with the BR hypothesis. However, only 16% of its households use organic manure, which according to BR should have become an important technology for soil fertility maintenance in this country. Moreover, agro-chemicals are used by only 3% of farmers. Malawi is doing far better with respect to seeds and fertilizers than with respect to crop intensity, irrigation, organic fertilizer and pesticides. Malawi appears to be a major puzzle for the BR framework, according to which we should have seen higher levels of all intensification levels, rather than the very uneven pattern across them.

In terms of inputs, Ethiopia appears to have a more even performance than Malawi. In Ethiopia, 53% of its farmers use organic fertilizer, 41% use inorganic fertilizer, and 18% and 23% use improved seeds and agrochemicals, respectively. Ethiopia has a strong agricultural extension system and also subsidizes fertilizer. In terms of the BR intensification variables, Ethiopia conforms well to BR.

Niger does very well in terms of use of organic fertilizer too, at 48% of the farmers. This may be because in the arid areas cattle herding is very important and manure more easily available, while in Ethiopia it may be caused by the widespread use of animal draft. However, Niger’s chemical fertilizers are used only by 18% of farmers, and the use of improved seeds and agrochemicals are also very low. The low use of improved seeds in Niger is likely to be associated with the unavailability of significantly improved varieties of sorghum and millet in the Sahel.

Forty-one percent of households in Nigeria use inorganic fertilizer and 34% use agro-chemicals. However, the use of organic fertilizer is the lowest among the countries, at only 3%. This low use in the country with the lowest agro-ecological potential per person is again inconsistent with BR.

Tanzania’s use of the four inputs varies between 12% for agro-chemicals and 18% for improved seeds. That the use of these inputs is low in the country with the highest agro-ecological potential per capita is consistent with BR.

In Uganda, the use of improved seeds is at 18%, while that of inorganic fertilizer is at only 3% of households. Organic fertilizer and agro-chemicals fall in between, at around 12%. Even though its agro-ecological potential per person is far lower than for Tanzania, it is doing worse than Tanzania in terms of inputs, again a challenge for BR.

## 6 Regression results

In this section, we report on (a) the estimates of the causal impact of AEP/ha and UG on population density and distances to road and markets, and (b) on a range of agricultural intensification variables. AEP and UG are exogenous to the conditions in the enumeration areas and, apart from issues of measurement error, should estimate causal links. As discussed, the regressions under (b) estimate the total impact of AEP and UG on the variables on the intensification variables, including the effect that goes via population density and market access.

For all variables, the individual observations are aggregated to their mean at the EA level. There are 1993 EAs located in six countries. The regressions are estimated in double log form, and, apart from the two variables of interest, AEP, UG and their interaction, include only country dummies.^24^ By doing so, only the within-country variations are used to estimate the equations and differences in policies, and other country-specific factors are therefore left out.

In Table 5, the R-squares for the three equations population density and distances to road and markets are between 0.12 and 0.14. Population density and road investments have responded over the past to AEP, but not the distance to markets. In absolute terms, the coefficient of AEP for distance to road is almost three times than that of population density. While road investment has been responsive to AEP, market distance has not, suggesting that factors other than AEP determine investments in, or emergence of, markets.

Urban gravity, on the other hand, does not affect population density, perhaps because the growth of urban areas has been too recent for population density to respond. But instead, it has a strong impact on distance to roads, with an elasticity of 0.31, more than twice as high as that of AEP. Market distance is also reduced for EAs subject to more urban gravity, suggesting that market investments respond to urban gravity.

It is therefore clear that both population and public investment in the past have responded significantly to AEP and UG, which is as we expected. Therefore, cross section regressions explaining any intensification variable (or any other agricultural variable that stems from a public or private decision), with population density and infrastructure variables, will lead to upwardly biased coefficients of the independent variables. As discussed, the problem can be overcome using panel data with fixed effects, as done in Binswanger et al. (1993).

Table 6 looks at area farmed, crop intensity and current fallow. The R-squares or Pseudo R-squares vary between 0.16 for crop and perennial area, to 0.77 for area under fallow. AEP does not influence any of the five variables, while UG affects all, except for the fallow variable. Own area, cropped area and crop and perennial area decrease with elasticities from −0.05 to −0.09, while crop intensity has a smaller absolute elasticity of 0.03. This is the only variable for which the interaction term of UG and AEP is statistically significant. The elasticity of AEP with respect to crop intensity at the mean of AEP is only 0.003, but still statistically significant. Unless there are left out variables with opposing impacts on these variables, these total impacts suggest that they are unresponsive to AEP in general, and therefore may also be unresponsive to population density. On the other hand, land areas decline with urban gravity, while crop intensity increases.

In Table 7, the share of land irrigated is unresponsive to either AEP or UG and seems to be determined by other factors, such as availability of canal or groundwater. However, AEP has a significant impact on all four technology variables, with the largest elasticity of 0.07 for inorganic fertilizer and the lowest one at 0.03 for organic fertilizer. As discussed in the introduction, this means that the regressions are not inconsistent with the BR predictions. Note also that the results are consistent with our constructed AEP measure being a valid proxy for the “true” underlying AEP.

The interpretation of these finding is that higher input use has significantly higher payoffs in areas of high AEP than of low AEP. This, of course, is well known, but it is interesting to see that our AEP variable and the household data can capture this effect. The estimated coefficients suggests that there is room in these total impacts for an impact via population density and market access. On the other hand, except for the use of improved seeds, UG has very little to do with use of inputs. This stands in contrast to the significant impact of UG on cropping intensity.

## 7 Summary and conclusions

### 7.1 New measures of agro-ecological potential and of urban gravity

This is the first paper to develop internationally comparable measures of agro-ecological potential and urban gravity. These measures impact positively on population densities, public investments in road and markets, and on some indicators of agricultural intensification.

We find that AEP per person ranks countries quite differently than with respect to AEP per ha. The AEP/ha of Uganda is by far the highest among the countries, the lowest being Niger, with Tanzania close to the average across countries. However, in terms of AEP per rural person, this is the highest in Tanzania, followed by Niger, and then only Uganda. The lowest potential per person is in Nigeria. These reversals of the measures of potential arise because of the sharply different population densities in the countries.

### 7.2 Descriptive results

Given the rise in population pressure in all these African countries, the improvements in infrastructure and the growing urban demand land use intensity, consistent with BR predictions, has reached permanent cropping in all of the countries. Fallow areas have virtually disappeared. Under permanent agriculture, high doses of organic and inorganic fertilizers are required to maintain or restore the soil nutrients taken out by the plants. Except for Malawi and Ethiopia, the proportion of households using chemical fertilizers is clearly too low to do so. Nor is this compensated for by the high proportion of households using organic fertilizer, which is relatively high only in Niger and Ethiopia. The BR theory also predicts that, under pressure from population growth and market access, irrigation investment and other modern technologies would be used more intensively to increase yields. However, these factors did not trigger significant irrigation investments, even in semi-arid areas where the payoff to irrigation is high. Unfortunately, we do not have data on other land investments, or on mechanization, to judge whether expected intensification responses have occurred with respect to these important investments. However, the descriptive analysis suggests that the BR impacts of population pressure and market access have triggered an inadequate response of the farming systems with respect to irrigation and technology use.

An additional inconsistency arises when comparing Tanzania with Malawi, with Tanzania having about 2.4 times the AEP/person as Malawi. Yet, cropping intensity is about the same and so is the intensity of use of manure. Use of agro-chemicals is more prevalent in Tanzania than in Malawi. The only area where Malawi has greater intensity of input use than Tanzania is in the use of inorganic fertilizers and improved seeds. In addition to being triggered by the forces of intensification, these higher uses are consistent with the long-standing effort of Malawi to increase the use of these two factors, including the significant subsidies that have been provided again in recent years.

As stressed all along, while the descriptive analysis can uncover apparent inconsistencies of cross-country patterns with the BR framework, the descriptive analysis provides no rigorous tests of it. First of all, there are variations in soils, crops and other biological variables that are likely to have a significant impact on the degree of intensification. These have been ignored so far. In addition, there are sharp differences in policies and infrastructure investments that have not been taken into account. It is therefore important that the theory be tested with panel data, where these other variations can be aggregated into fixed country effects.

### 7.3 Regression analysis

We found significant responses of population density and infrastructure, farming systems characteristics, farm technology and profits per ha to our measures of AEP and UG, and the signs are all according to expectations. However, there is a sharp divide between the nature of the impact of AEP and UG across the variables:

• AEP increases population density and road investment, but not distance to markets, while UG does not affect population density, but reduces both the distance to roads and to markets.
• AEP has no impact at all on key characteristics of the farming system, such as areas farmed, crop intensity and fallow areas, while UG reduces all area measures and increases cropping intensity.
• While neither AEP nor UG have an impact on irrigation investment, AEP affects the use of all four inputs, while UG only increases the use of improved seeds.

We have provided a few hints as to why the response patterns with respect to AEP and UG differ so significantly, but a full understanding will undoubtedly require more sophisticated research approaches. In terms of testing BR with respect to UG, we see that it increases crop intensity and improved seeds, but not the other technology variables, which does not provide much support for the operation of the BR predictions in Africa.

### 7.4 Implications

The facts described in this paper are only partially consistent with the BR framework. In particular, and in line with other findings in the literature, the use of organic and chemical fertilizers, except perhaps in Malawi and Niger, appears far too low to maintain soil fertility. Except for Ethiopia, this also applies to the use of organic fertilizers. In addition, investments in irrigation also seem to fall far short of what the high population densities and significant market access would require. This last finding is consistent with Headey and Jayne (2014), who stress that other investments, such as mechanization, also have responded inadequately to rising population pressure. The implication of these results, and of the observations of many other observers of African agriculture, is that the process of intensification over much of these African countries appears to have been less beneficial to farming systems and farmers than what could have been expected according to the BR hypothesis.

## Figures and Tables

**Table 1:** Countries’ Endowments.

	ETH	MWI	NER	NGA	TZA	UGA	Total
1. Value of agro-ecological potential (US$/ha)	691.2	999.1	478.7	657.0	786.4	1877.9	739.6
2. Agroecological potential per person	396.7	547.6	792.4	301.0	1313.5	703.7	393.8
3. Rural population density (pers./sq. km) (2005)	174.2	182.5	60.4	218.3	59.9	266.9	187.8
4. UG*	7.4	169.3	22.8	134.6	30.1	63.6	82.9
5. Distance (in Kms) to the nearest major road	14.4	10.6	11.5	16.0	17.8	7.9	15.3
6. Households’ distance (in Kms) to the nearest market	64.5	7.7	56.3	70.1	70.4	31.6	66.3

* UG travel time in hours to cities with 500 K population.

**Table 2:** Households’ characteristics.

	ETH	MWI	NER	NGA	TZA	UGA	Total
1. Head’s age		43.0	44.5	51.2	48.5	45.8	48.8
2. Share of female head		0.2	0.1	0.1	0.2	0.3	0.2
3. Gross income from crop per ha (US$/ha)		500.5	179.6	1144.6	519.9	495.3	983.4
4. Gross household income = Ag wage + Non-ag. wage + Crop + Livestock + Self employment + Transfer (US$)		622.2	1235.7	1413.9	1072.8	1164.4	1333.6
5. Gross income per capita (US$/pc)		130.99	181.19	234.87	188.54	192.78	227.42
6. Poverty headcount ratio below PPP $1.25/day (2005)		75.2	40.8	65.5	91.5	52.5	66.6

Data on income and consumption for ETH not available. As in Deininger, Xia, and Savastano income figures are doubtful for Nigeria where there are some data issues (Oseni et al., 2014) therefore the descriptive statistics should be interpreted carefully.

**Table 3:** Land and fallow.

	ETH	MWI	NER	NGA	TZA	UGA	Total
1. Area owned (ha)	1.2	0.68	4.5	1.1	2.41	1.8	1.3
2. Area operated (ha)	1.3	0.74	5.1	1.4	2.45	2.0	1.6
3. Gross cropped area (ha)	0.6	0.74	5.8	1.6	2.03	2.4	1.5
4. Net cropped area (ha)	0.3	0.67	4.9	1.3	1.95	1.0	1.1
5. Crop intensity	1.21	1.02	1.19	1.23	1.07	1.89	1.23
6. Proportion of current fallow	NA	0.0	0.1	0.0	0.3	0.1	0.0
7. Proportion of past fallow in current fallow	NA	0.01	0.03	NA	0.08	0.05	0.01

**Table 4:** Irrigation and technology by country.

	ETH	MWI	NER	NGA	TZA	UGA	Total
1. Irrigated area (ha)	0.016	0.003	0.036	0.033	0.045	0.02	0.029
2. Dummy improved seeds	0.18	0.61	0.03	NA	0.18	0.18	0.09
3. Dummy inorganic fertilizer	0.41	0.76	0.18	0.41	0.16	0.03	0.38
4. Dummy organic fertilizers	0.53	0.16	0.48	0.03	0.17	0.12	0.25
5. Dummy agro-chemicals	0.23	0.03	0.07	0.34	0.12	0.11	0.27

**Table 5:** Population density and infrastructure.

	(1)	(2)	(3)
	Log Pop. Dens.	Log Dist. To Road	Log Distance to Mrkt
Log Value of AEP $/ha	0.056∗	−0.146∗∗∗	0.001
UG^a^	0.066	−0.309∗∗∗	−0.061∗
Interaction Log UG and Log AEP	−0.001	0.024∗∗∗	−0.006
Country dummy ETH	0.393∗∗∗	−0.274∗∗	−0.325∗∗∗
Country dummy MWI	0.289∗∗	−0.069	−1.960∗∗∗
Country dummy NER	−0.947∗∗∗	−0.670∗∗∗	−0.705∗∗∗
Country dummy TZA	−0.971∗∗∗	−0.219∗	−0.376∗∗∗
Country dummy UGA	0.508∗∗∗	−0.472∗∗∗	−0.935∗∗∗
Constant	4.092∗∗∗	3.453∗∗∗	4.292∗∗∗

Observations	1993	1993	1993
R-squared	0.118	0.136	0.122

Nigeria is the baseline for the country dummy.

a UG: travel time negative exponential, with borders restriction to cities with 50.

*** *p* < 0.01.

** *p* < 0.05.

* *p* < 0.1.

**Table 6:** Land areas and intensification.

	OLS				Tobit
	Log Own Area	Log Crop Area	Log Crop and Perennial Area	Crop intensity	Proportion of land under current fallow^b^
Log Value of AEP $/ha	0.016	0.006	−0.002	0.001	−0.002
UG^a^	−0.086∗∗∗	−0.054∗∗	−0.062∗∗∗	0.029∗∗∗	0.0005
Interaction Log UG and Log AEP	0.003	−0.001	0.000	−0.004∗∗∗	−0.001
Country dummy ETH	−0.067	−0.602∗∗∗	−0.161∗∗∗	−0.086∗∗∗	
Country dummy MWI	−0.094∗∗∗	−0.185∗∗∗	−0.229∗∗∗	−0.102∗∗∗	0.122∗∗∗
Country dummy NER	0.761∗∗∗	0.801∗∗∗	0.761∗∗∗	−0.019	0.131∗∗∗
Country dummy TZA	0.291∗∗∗	0.166∗∗∗	0.128∗∗∗	−0.090∗∗∗	0.295∗∗∗
Country dummy UGA	0.238∗∗∗	0.121∗∗∗	0.197∗∗∗	0.205∗∗∗	0.248∗∗∗
Constant	0.694∗∗∗	0.848∗∗∗	0.934∗∗∗	0.796∗∗∗	−0.250∗∗∗

Observations	1993	1993	1993	1993	1750
R-squared	0.256	0.320	0.159	0.158	0.771

Elasticity of AEP taking account of both the linear and the interaction term				−0.0032	
P-value				0.543	
Elasticity of UG taking account of both the linear and the interaction term				0.0241	
P-value				0.001	

Nigeria is the baseline for the country dummy.

a UG: travel time negative exponential, with borders restriction to cities with 50.

b Information on Proportion of land under current fallow is NA in ETH.

*** *p* < 0.01.

** *p* < 0.05. ^*^ *p* < 0.1.

**Table 7:** Irrigation and technology variables, Tobit regression.

Variables	Tobit Regressions				Probit Regression
	Share of Land irrigated	Share organic fertilizer	Share inorganic fertilizer	Share agro-chemicals	Share of Improved seeds b
Log Value of AEP $/ha	−0.054	0.030∗∗∗	0.071∗∗∗	0.048∗∗	0.059∗∗∗
UG^a^	−0.169	−0.021	−0.027	−0.038	0.122∗∗
Interaction Log UG and Log AEP	0.021	0.000	0.001	−0.003	−0.019∗∗∗
Country dummy ETH	0.457∗	0.946∗∗∗	0.182∗∗∗	−0.237∗∗∗	−0.720∗∗∗
Country dummy MWI	−0.302∗	0.450∗∗∗	0.455∗∗∗	−0.731∗∗∗	
Country dummy NER	−0.325	0.848∗∗∗	−0.245∗∗∗	−0.533∗∗∗	−0.626g
Country dummy TZA	−0.022	0.312∗∗∗	−0.484∗∗∗	−0.590∗∗∗	−0.750∗∗∗
Country dummy UGA	−0.444∗∗	0.264∗∗∗	−0.818∗∗∗	−0.448∗∗∗	−0.675∗∗∗
Constant	−0.879∗	−0.465∗∗∗	−0.081	0.070	

Observations	1993	1993	1993	1993	1633
R-squared	0.0356	0.486	0.185	0.0917	0.0256

Nigeria is the baseline for the country dummy in all other regressions.

a UG: travel time negative exponential, with borders restriction to cities with 50.

b Regressions on Improved seeds does not include NGA as the variable is not available. MWI is the baseline in this case.

*** *p* < 0.01.

** *p* < 0.05.

* *p* < 0.1.

## Footnotes

1 In less densely populated countries and regions, it is still possible to maintain farm sizes as emphasized by Headey and Jayne (2014).

2 In addition to intensification, farmers can diversify into cash crops and buy food, or they can migrate. These opportunities are better in an open economy than in a closed one.

3 A study of agricultural intensification in Africa found signs of involution only in 2 of 10 locations they studied (Turner et al., 1993).

4 We leave out the smaller cities, as their income as measured via light emissions could be affected by the agro-ecological potential of the zone in which they sit, making them endogenous to the system analyzed.

5 As a proxy of light intensity, we used the sum of nighttime lights recorded in 2009. Input values ranging from 0 to 63 indicate average intensity of light observations, regardless of frequency of observation. Ephemeral events such as lightning strikes and fires have been discarded. The satellite source is DMSP F16, inter-calibrated for comparison between years. The range of 0–63 refers to the pixel-level value (the source data are gridded at 30 arc seconds). The variable we are using is aggregated at the 5 arc minute block level (resolution of SPAM, GAEZ and other harvest choice variables), which would include many pixels from the lights data.

6 There are some parallels of the BR model with the Induced Innovation model (Hayami and Ruttan, 1985) in that under BR, it is population density and market access that push agricultural intensification, including new technological innovations and institution that have to underpin it, while under the induced innovation model, it is technologies, land/labor ratios and institutions that adapt. However, each of the theories cannot explain what the other theory explains, and vice versa.

7 Women had secure access to unimproved land for their subsistence production, but once a parcel was improved via erosion control, would lose such access.

8 They also develop a general model of intensification that can accommodate the impact on per capita income of land expansion, intensification, reduced rural fertility rates, and diversification into non-farm activities.

9 The Global Agro-Ecological Zones website can be found here: http://www.fao.org/nr/gaez/en/

10 For the coordinates of the community to be matched to the geographic units of the GAEZ data, we calculate the central point of each of the enumeration areas, using the geo-location of the households in the EA. We select the corresponding grid cell from the IIASA-FAO data set, as well as the adjacent grid cells. We average across such geographic units by weighing the values for the adjacent grid cells by their Euclidian distance from the central point of the EA for which we calculate the AEP.

11 If there is little use of fertilizer or manure, soil fertility has to be restored by leaving land fallow. The fallow requirement may be one year or more. The fallow adjustment converts the model result to the average number of growing years in the crop-fallow cycle.

12 In principle, the AEP should include livestock production possibilities, but such data do not exist.

13 The GAEZ data also include a low input level and a high input level. There are many countries in Africa in which the low input level is no longer practiced. The high input level is only practiced in the commercial farm sectors for example of South Africa or Kenya and therefore does not reflect what smallholders do or can aspire to.

14 Another way to aggregate across crops is to use calories per kilogram of each crop, and then aggregate them as discussed in the next paragraph. However, the market value of calories from different crops is very different, as exemplified by calories from tubers relative to calories from grains. Moreover, farmers are not interested in the calories they can produce per ha, but in the revenues that they generate.

15 Harvest Choice data are based on calculations from data from the Center for International Earth Science Information Network (CIESIN), Columbia University; International Food Policy Research Institute (IFPRI); The World Bank; and Centro Internacional de Agricultura Tropical (CIAT), 2004. We cannot use the population computed from LSMS-ISA as the EAs have been chosen with probability proportional to their population at the last census giving preference to EAs with higher population. If we had aggregated our measure of population at the EA level, low population density areas would have been underrepresented.

16 If GDP data are flawed, they may be a superior measure of economic activity at the national level. The authors present estimates across countries and over time of reported GDP and light intensity, and also present an estimator of GDP which optimally combines the two.

17 The data source is the DMSP F16, the U.S. Air Force’s research project called the Defense Meteorological Satellite Program (DMSP), established in 1960. Since 1994, DMSP have produced a time series of annual cloud-free composites of DMSP nighttime lights. Together with the NGDC – EOG (the National Geographic Data Center – Earth Observation Group).

18 We choose cities with populations over 500,000 at the current time, because smaller cities often are centers of agricultural services and markets, and therefore their population is influenced by the AEP of the surrounding area, which makes it endogenous.

19 Ruthenberg, 1980a, Ruthenberg, 1980b R-value RV = N ∗ 100/(N + F), where N is net cropped area (also called cultivated area) and F is fallow area.

20 This seems low. Note that famers will obtain less than this value because most of them are far from intermediate input levels used to calculate the AEP.

21 Note that the AEP only takes account of the first season, and that Uganda in many areas is able to grow two rain-fed crops, and therefore has by far the highest cropping intensity (Table 6). Therefore, the real advantage of Uganda is even more striking.

22 For interpretation of color in Map 1 and 2, the reader is referred to the web version of this article.

23 Recall that this is the average population density across the EAs in each sample, which will be higher than the rural population density reported in national statistics, as it comes from a sample of EAs chosen with probability proportional to population size, rather than from all EAs in the Census.

24 Square terms were insignificant for all dependent variables and therefore were dropped it from the regression.
