# Supplementary material for: Agricultural intensification: The status in six African countries
Source: Food Policy. 2017 Feb;67:26–40. doi: 10.1016/j.foodpol.2016.09.021 (PMC5384439; doi:10.1016/j.foodpol.2016.09.021)
Supplement: Supplementary data 1 [file mmc1.docx]

# Online Annex on results for agro-ecological zones

In this Annex, the results are provided for the seven agro-ecological zones that are found in these countries. They are tropical areas falling into the following zones: warm arid, warm semi-arid, warm sub-humid, warm humid, cool semi-arid, cool sub-humid and cool humid.

The cool humid tropics have always been considered to be very fertile. In Table A1, it is therefore not surprising that the AEP/ha is by far the highest ($1161). But because these areas also have high population densities, the AEP per person is down to $489, less than half the AEP/ha, and only about 100 dollars above the average across countries. While the warm arid tropics have by far the lowest AEP/ha, their AEP/person is the highest among all the AEZs, at $1109, primarily on account of its very low rural population density of 14 persons/km2. These findings suggest that migration and population growth over the past have far more than equalized the chances of people living in the most challenging climate environments with those in the better ones, so that they actually face lower agro-ecological pressure than the better endowed climate zones. The warm arid areas are followed by the warm semi-arid areas, another challenging climate zone, at $627 per person. The warm sub-humid areas and the cool semi-arid areas have the lowest agricultural potential per person AEPD, at only $304. Urban gravities vary even more across AEZs than countries, with the lowest one found in the warm arid areas, at only 0.1, and the highest in the warm humid areas, at 299.

**Annex table 1: Endowments by tropical agro-ecological zone (AEZ)**

|  | **Warm arid** | **Warm semiarid** | **Warm sub-humid** | **Warm humid** | **Cool semi-arid** | **Cool sub-humid** | **Cool humid** | **TOTAL** |
| --- | --- | --- | --- | --- | --- | --- | --- | --- |
| 1. Value of agroecological potential (US$/ha) | 152.4 | 585.0 | 732.6 | 936.0 | 350.3 | 867.1 | 1161.5 | 739.6 |
| 1. AEP per person (US$/person) | 1109.5 | 627.0 | 303.6 | 325.9 | 304.4 | 508.6 | 489.3 | 393.8 |
| 1. Rural population density(pers./sq. km) (2005) | 13.7 | 93.3 | 241.3 | 287.2 | 115.1 | 170.5 | 237.4 | 187.8 |
| 1. Average growing period (days) | 48.3 | 124.3 | 234.1 | 298.9 | 141.9 | 224.8 | 277.2 | 208.6 |
| 1. HH distance in (KMs) to nearest major road | 30.7 | 21.3 | 14.5 | 7.7 | 16.5 | 12.6 | 15.3 | 15.3 |
| 1. HH Distance in (KMs) to nearest market | 54.2 | 64.3 | 75.5 | 48.2 | 55.9 | 61.9 | 70.8 | 66.3 |

(*) UG travel time in hours to cities with 500K population.

*Source: Authors’ computation from LSMS-ISA surveys*

Annex Table 2 shows that is the highest in the warm arid area (3.08 ha) where population density is lowest, and the lowest in the warm humid areas where population density is highest. In this zone, the cropping intensity is also the highest, at 1.59, while it varies between 1.13 and 1.31 across the other climate zones. The proportion of land area fallowed is minimal everywhere and cannot possibly restore soil fertility.

**Annex Table 2: Land and fallow by AEZ**

|  | **Warm arid** | **Warm semiarid** | **Warm sub-humid** | **Warm humid** | **Cool semiarid** | **Cool subhumid** | **Cool humid** | **TOTAL** |
| --- | --- | --- | --- | --- | --- | --- | --- | --- |
| Area owned (ha) | 3.1 | 1.8 | 1.1 | 1.0 | 1.2 | 1.5 | 1.2 | 1.3 |
| Gross cropped area (ha) | 3.9 | 2.2 | 1.6 | 1.6 | 0.7 | 1.0 | 0.7 | 1.5 |
| Net crop area (ha | 3.0 | 1.9 | 1.2 | 0.8 | 0.4 | 0.7 | 0.4 | 1.1 |
| Crop intensity | 1.2 | 1.1 | 1.2 | 1.6 | 1.2 | 1.2 | 1.3 | 1.2 |
| Past fallow area (ha) | 0.07 | 0.01 | 0.02 | 0.16 | 0.01 | 0.03 | 0.08 | 0.04 |
| Prop. of current fallow area in current crop and fallow area | 0.003 | 0.004 | 0.01 | 0.02 | 0.003 | 0.02 | 0.01 | 0.01 |

*Source: Authors’ computation from LSMS-ISA surveys*

In Table 3, the area of land irrigated is by far the highest in the warm arid areas (0.11ha). This is not surprising because the payoff to irrigation is higher, the dryer the climate. In all other climate zones it is around 0.01-0.05 ha. This is also not surprising in the cool or warm humid and sub-humid areas, because the payoff to irrigation is lower in such areas than in more arid zones. What is surprising is that the cool and the warm semi-arid tropics have such low irrigation levels, as here the payoffs to irrigation are higher than in more humid areas. Irrigation, with the promise of a secure crop in the first season and a crop in the second season, should long have been a favored investment for farmers in these zones. Even if groundwater resources in Africa are lesser than in South and East Asia, for some farmers they are still available. Many of these could have used bore-wells to install irrigation.

That irrigation, even in the semi-arid and arid zones where payoffs to irrigation are very high, is so low despite growth in population and urban demand, suggests that farmers have not responded to these trends by increasing irrigation, as the BR framework would predict. Is it possible that this lack of response it caused by exceptionally poor availability of groundwater, which farmers could have tapped via bore-wells?

Improved seed use is significantly higher in the cool areas than in the warm areas (11-25%, versus 1.2-6.9%). A similar difference arises for organic fertilizer, where the cool areas have uses varying between 56% and 61%, while for the warm areas they vary between 3% and 13%. In warm areas, it is very difficult to accumulate soil organic matter, which decays rapidly when exposed to heat, while in cool or cold areas it is far easier to do so. The poor returns to organic fertilizer may therefore be a major barrier to intensification in the warm areas, compared with the cool areas. There are hints in the literature that this may indeed be the case, with consistency of intensification with BR better in the cooler areas than in warm areas.

The use of chemical fertilizers across the cool zone and the warm semi-arid zone is much higher than in the remaining three warm zones (40% to 53%, versus 6% to 26%). This may be related to the higher use of improved seeds in the cool areas already discussed, although high fertilizer use and low seed use go together in the warm semi-arid zones. The warm areas, other than the semi-arid and arid ones, have soils that are low in cation-exchange capacity, which limits the payoff to chemical fertilizer**.**

**Annex Table 3: Irrigation and Technology by AEZ**

|  | **Warm arid** | **Warm semiarid** | **Warm sub-humid** | **Warm humid** | **Cool semiarid** | **Cool sub-humid** | **Cool humid** | **TOTAL** |
| --- | --- | --- | --- | --- | --- | --- | --- | --- |
| 1. Irrigated area (ha) | 0.11 | 0.05 | 0.03 | 0.01 | 0.03 | 0.02 | 0.01 | 0.03 |
| 1. Dummy using organic fertilizers | 0.11 | 0.13 | 0.03 | 0.03 | 0.61 | 0.56 | 0.58 | 0.24 |
| 1. Dummy using improved seeds | 0.01 | 0.02 | 0.03 | 0.07 | 0.11 | 0.25 | 0.18 | 0.09 |
| 1. Dummy using inorganic fertilizer | 0.07 | 0.50 | 0.26 | 0.06 | 0.40 | 0.53 | 0.46 | 0.38 |
| 1. Dummy using agro-chemicals | 0.14 | 0.38 | 0.27 | 0.06 | 0.15 | 0.27 | 0.31 | 0.27 |

*Source: Authors’ computation from LSMS-ISA surveys*
